# Profiling and production of hemicellulases by thermophilic fungus *Malbranchea flava* and the role of xylanases in improved bioconversion of pretreated lignocellulosics to ethanol

**DOI:** 10.1007/s13205-015-0325-2

**Published:** 2016-01-14

**Authors:** Manju Sharma, Chhavi Mahajan, Manpreet S. Bhatti, Bhupinder Singh Chadha

**Affiliations:** 1 Department of Microbiology, Guru Nanak Dev University, Amritsar, Punjab 143005 India; 2 Department of Botanical and Environmental Sciences, Guru Nanak Dev University, Amritsar, Punjab India

**Keywords:** Xylanases, Xylan-debranching accessory enzymes, Secretome analysis, Response surface methodology (RSM), Saccharification of lignocellulosics

## Abstract

This study reports thermophilic fungus *Malbranchea flava* as a potent source of xylanase and xylan-debranching accessory enzymes. *M. flava* produced high levels of xylanase on sorghum straw containing solidified culture medium. The optimization of culture conditions for production of hemicellulases was carried out using one factor at a time approach and Box–Behnken design of experiments with casein (%), inoculum age (h) and inoculum level (ml) as process variables and xylanase, β-xylosidase, acetyl esterases and arabinofuranosidase as response variables. The results showed that casein concentration between 3.0 and 3.5 %, inoculum age (56–60 h) and inoculum level (2–2.5 ml) resulted in production of 16,978, 10.0, 67.7 and 3.8 (U/gds) of xylanase, β-xylosidase, acetyl esterase and α-l-arabinofuranosidase, respectively. Under optimized conditions *M. flava* produced eight functionally diverse xylanases with distinct substrate specificity against different xylan types. The peptide mass fingerprinting of 2-D gel electrophoresis resolved proteins indicated to the presence of cellobiose dehydrogenase and glycosyl hydrolases suggesting the potential of this strain in oxidative and classical cellulase-mediated hydrolysis of lignocellulosics. Addition of xylanase (300 U/g substrate) during saccharification (at 15 % substrate loading) of different pretreated (acid/alkali) substrates (cotton stalks, wheat straw, rice straw, carrot grass) by commercial cellulase (NS28066) resulted in 9–36 % increase in saccharification and subsequent fermentation to ethanol when compared to experiment with commercial enzyme only. High ethanol level 46 (g/l) was achieved with acid pretreated cotton stalk when *M. flava* xylanase was supplemented as compared to 39 (g/l) with xylanase without xylanase addition.

**Electronic supplementary material:**

The online version of this article (doi:10.1007/s13205-015-0325-2) contains supplementary material, which is available to authorized users.

## Introduction

Hemicellulose is the second most abundant biopolymer in plant cell wall after cellulose which exists as *O*-acetyl-4-*O*-methylglucuronoxylan in hardwoods and as arabino-4-*O*-methylglucuronoxylan in softwoods, while xylan in grasses and annual plants are typically arabinoxylans consisting of a β-1,4-linked backbone of d-xylopyranosyl residues to which α-l-arabinofuranosyl (araf) residues are linked at C-3 and C-2 (Scheller and Ulvskov 2010). Owing to its complexity, the complete hydrolysis of xylan requires the action of main and side chain cleaving enzymes including endo-β-1, 4-xylanase (E.C. 3.2.1.8), β-D-xylosidase (E.C. 3.2.1.37), α-l-arabinofuranosidase (E.C.3.2.1.39), acetyl xylan esterase (E.C. 3.1.1.72) and ferulic or *p*-coumaric acid esterase (E.C.3.2.1.73) (Shallom and Shoham 2003). In recent years, xylanases have received a great deal of research attention particularly because of their biotechnological potential in food, feed, and pre-bleaching of pulps in paper industries. Each of these applications do require xylanases with distinct physico-chemical properties in terms of their mode of action, substrate specificity, pH, temperature optima, etc. (Polizeli et al. 2005). Recent reports on xylanases (derived from recombinant *T. reesei* Multifect^®^) suggest important role of these xylanases in enhancing the capability of the cellulases for hydrolysis of pretreated substrates (Hu et al. 2013). However, owing to increasing biotechnological importance of thermostable xylanases, there is a need to identify novel and catalytic efficient sources of xylanases for their role in improving these processes. Few of the important thermophilic fungal strains (*Thermomyces lanuginosus*, *Myceliophthora* sp., *Melanocarpus albomyces*, *Paecilomyces thermophila*) as sources of thermostable xylanases have been reported in recent past (Saraswat and Bisaria 1997; Sonia et al. 2005; Yang et al. 2006; Badhan et al. 2007). This study reports production of functionally diverse xylanases and xylan-debranching accessory enzymes by a novel thermophilic fungal strain *M. flava* isolated from composting soils (Sharma et al. 2008). The preliminary studies showed that culture produced appreciable levels of xylanase, however, for commercial application it is desirable to improve titres of xylanase and xylan-debranching accessory enzymes and, therefore, culture conditions for optimization of enzyme production using solid-state fermentation was studied. Furthermore, culture extracts were also used for profiling multiple xylanases, β xylosidases, α-l-arabinofuranosidases and acetyl esterases as well as secretome analysis using peptide mass fingerprinting approaches. The role of *M. flava* xylanases in enhancing saccharification of differently pretreated substrates for subsequent fermentation to ethanol was also examined.

## Materials and methods

### Microorganism and growth conditions

A thermophilic fungus isolated from composting soil and identified as *M. flava* (Sharma et al. 2008) was grown and maintained on malt extract agar (pH 7.0) with following composition (g/l): malt extract 20 and agar 20. The fungus was cultured at 45 °C for 10 days and stored at 4 °C.

### Solid-state fermentation (SSF) for enzyme production by *M. flava*

The production of xylanases and xylan-debranching enzymes was carried out using solidified culture medium in Erlenmeyer flasks (250 ml). The culture medium comprised of ground rice straw (5 g) as carbon source and 17.5 ml of basal medium with following composition (% w/v), ammonium acetate, 0.6; (NH)_2_SO_4_, 0.3; KH_2_PO_4_, 0.240; MgSO_4_·7H_2_O, 0.048; CaCl_2_·2H_2_O, 0.010. Prior to sterilization, the pH of the medium was adjusted to 7.0 with 1 N NaOH. The culture medium was inoculated with 2 ml of spore suspension 2.5 × 10^7^ (spores/ml) and incubated for 6 days in water-saturated atmosphere at 45 °C. Thereafter, the enzyme was harvested by adding 50 ml sodium citrate buffer (50 mM, pH 6.5) to the flasks and stirred mildly for 1 h at 40 °C. The fermented slurry was filtered through muslin cloth and centrifuged at 8000×*g* for 20 min. The resultant extract was used for enzyme assays and secretome profiling. Effect of process variables on enzyme production was studied using one factor at a time (OFAT) experiments. The effect of different carbon sources (rice straw, wheat bran, sorghum straw, wheat straw, bagasse, corn cobs, groundnut shell), nitrogen sources (peptone, malt extract, beef extract, tryptone, casein, soybean meal, corn steep liquor and inorganic sources ammonium sulphate, NH_4_NO_3_, (NH_4_)_2_HPO_4_, sodium nitrate, ammonium acetate, urea), inoculum age (0–96 h), inoculum level (1–6 ml), temperature (35–50 °C), pH (4.0–10.0), moisture content (55–80 %), on production of xylanase were studied.

### Box–Behnken design for process optimization

Based on OFAT experiments, nitrogen (% casein), inoculum age (h), and inoculum level (ml) were identified to influence enzyme production significantly and were chosen as independent process variable for optimization by Response Surface Methodology (RSM) using Box–Behnken design of experiments employing 3 levels (−1 as minimum; 0 for center; and +1 as maximum) for each independent variable. The casein concentration was studied between 0.5 and 3.5 % w/v, inoculum age between 0 and 72 h and inoculum level between 2.0 and 4.0 ml. A total of 17 experimental runs were performed in different flasks with five replicates having all the three variables at their central coded values as given in Table 1. The mathematical relationship between independent variable (casein concentration, inoculum age and inoculum level) and dependent variables X (xylanase, β-xylosidase, acetyl esterases and arabinofuranosidase) was generally approximated by the quadratic model as given in Eq. (1).

1 $$Y = b_{\text{o}} + \sum\limits_{n = 1}^{n} {b_{n} X_{n} } \, + \sum\limits_{n = 1}^{n} {b_{nn} X_{n}^{2} } + \sum\limits_{{}}^{n} {\sum\limits_{n = 1}^{n} {b_{nm} X_{n} X_{m} } }$$

where *Y* is the predicted response, *b*_o_, *b*_*nn*_ and *b*_*nm*_ are the linear, quadratic and interaction coefficients, respectively, and n is the number of independent variables. The *X*_*n*_ and *X*_*m*_ are the coded values of the independent variables as per CCD. Analysis of variance (ANOVA) was performed to determine the significant difference (*p* ≤ 0.05) in responses under different conditions. The model fitting was checked from analysis of variance (ANOVA) table using *F* values, degree of freedom (*df*), lack of fit, coefficient of variance (CV%) and coefficient of determination (*R* ^2^). These runs were conducted in randomized manner to guard against systematic bias. The optimized conditions were validated at the optimum level to check the model predictability. Statistical software (Design-Expert v 8.0.7, Stat-Ease Inc., USA) was used to obtain optimal working parameters and to generate response surface graphs.

**Table 1:** Box–Behnken design along with actual and predicted values of xylanase, β-xylosidase, acetyl esterase (AE) and α-l-arabinofuranosidase

Std	Independent variables						Response variables			
	A	B	C	A: Casein	B: Inocul. Age	C: Inocul. Level	Xylanase (U/gds)	β-xylosidase (U/gds)	Acetyl esterase (U/gds)	α-l-Arabino-furanosidase (U/gds)
	Coded level			Actual level			Actual	Actual	Actual	Actual
				%	h	ml				
1	−1	−1	0	0.5	0	3	9700	5.91	26.1	1.98
2	1	−1	0	3.5	0	3	12,140	5.31	50.1	3.10
3	−1	1	0	0.5	72	3	11,440	5.43	29.7	2.42
4	1	1	0	3.5	72	3	14,920	9.52	65.5	3.60
5	−1	0	−1	0.5	36	2	10,810	6.40	35.6	2.50
6	1	0	−1	3.5	36	2	16,390	9.49	69.8	3.40
7	−1	0	1	0.5	36	4	13,220	5.74	37.0	2.40
8	1	0	1	3.5	36	4	12,290	7.90	62.7	3.60
9	0	−1	−1	2.0	0	2	12,890	8.47	51.8	2.59
10	0	1	−1	2.0	72	2	15,460	10.00	51.5	3.50
11	0	−1	1	2.0	0	4	11,650	7.39	41.4	2.00
12	0	1	1	2.0	72	4	14,380	9.23	55.4	3.58
13	0	0	0	2.0	36	3	14,220	9.00	51.9	2.92
14	0	0	0	2.0	36	3	15,050	10.00	55.6	3.46
15	0	0	0	2.0	36	3	15,920	9.16	48.2	2.68
16	0	0	0	2.0	36	3	16,040	10.00	49.4	3.40
17	0	0	0	2.0	36	3	15,050	10.00	54.7	3.54

### Enzyme assay

Xylanase activity was estimated according to method described by Bailey et al. (1992). The assay mixture contained 1.8 ml of 1 % (w/v) birch wood xylan (Sigma, X-0502) as substrate (prepared in 0.05 M sodium citrate buffer pH 6.5) and 0.2 ml suitably diluted enzyme was incubated at 50 °C for 5 min. The reaction was stopped by adding 3 ml dinitrosalicylic acid (DNS) reagent and the contents were boiled for 10 min. The developed color was read at 540 nm using Novaspec II spectrophotometer (Pharmacia). The amount of reducing sugar liberated was quantified using xylose standard. One unit of xylanase activity was defined as the amount of enzyme required to release 1 µmol of xylose equivalents per minute. For xylan-debranching enzymes, the substrates used were (3 mM) of *p*-nitrophenyl acetate, *p*-nitrophenyl-β-d-xylopyranoside and *p*-nitrophenyl-α-l-arabinofuranoside for the assay of acetyl xylan esterase, β-xylosidase and α-l-arabinofuranosidase activities, respectively. For estimation of acetyl esterase, the reaction mixture (150 µl) containing appropriately diluted enzyme (25 µl) and substrate (125 µl) prepared according to Mastihuba et al. (2002) was incubated at 50 °C for 30 min in dark. For determination of β-xylosidase and α-l-arabinofuranosidase activities the reaction mixture comprising of appropriately diluted enzyme (25 µl), 0.05 M sodium acetate buffer pH 5.0 (50 µl) and substrate (25 µl) was incubated at 50 °C for 30 min. The reaction was stopped by adding 100 µl of NaOH–glycine buffer (0.4 M, pH 10.8) and developed color was read at 405 nm using ELISA Reader (BIORAD). The amount of p-nitrophenol released was quantified from the pNP standard. One unit of enzyme activity was expressed as the amount of enzyme required to release 1 µmol of *p*-nitrophenol under assay conditions.

### Electrophoresis and isoelectric focusing

The enzyme samples obtained by culturing *M. flava* under optimized culture conditions was desalted using ultra-filtration Amicon cell fitted with PM-10 membrane (10 kDa cutoff). The protein (70 µg) was fractionated by native-polyacrylamide gel electrophoresis (PAGE) using 7.5 % resolving gel with 4 % stacking gel using Mini-Protean II system (Bio-Rad). Similarly, samples were resolved by isoelectric focusing (IEF) which was performed according to the instructions provided by Novex (Invitrogen, Life Sciences, USA) using a 5 % acrylamide gel containing 2.4 % narrow range pH range (3–5) ampholine carrier servalyte (SERVA, Germany). The cathode buffer contained 0.35 % (w/v) arginine and 0.29 % (w/v) lysine, whereas 10 mM phosphoric acid was used as anode buffer. IEF was carried out for 1 h each at constant 100 and 200 V followed by 500 V for 30 min (Badhan et al. 2004). After fractionating the proteins on IEF, the gel in each lane was sliced (1.25 mm thickness). Each slice was incubated in 500 µl sodium citrate buffer (50 mM, pH 6.0) for 72 h at 4 °C. The eluted protein in each fraction was assayed for endoxylanase against birch wood xylan (BWX), rye arabinoxylan (RAX), wheat arabinoxylan (WAX) and 4-*O*-methyl glucuronoxylan (MGX) (Badhan et al. 2004).

### Activity staining

Xylanase activity in PAGE and IEF gels was detected by overlaying 1 % agarose replica containing RBB-Xylan (Badhan et al. 2004). Upon completion of electrophoresis, the gels were incubated in sodium citrate buffer (50 mM, pH 6.0) for 30 min and then overlaid on RBB-Xylan containing replica gel for 30–60 min at 50 °C. To avoid band diffusion, these gels were dried at 60 °C.

### Detection of β-xylosidases and α-l-arabinofuranosidases using 4-methylumbelliferyl xylopyranoside and α-l-arabinofuranoside

β-Xylosidase and α-l-arabinofuranosidase activities in the PAGE resolved gels were detected with 4-methylumbelliferyl-β-d-xylopyranoside and 4-methylumbelliferyl α-l-arabinofuranoside (10 mM) as respective substrates (prepared in 50 mM sodium citrate buffer pH 6.0). Upon completion of electrophoresis, the gels were incubated in sodium citrate buffer (50 mM, pH 6.0) for 30 min and then the substrate solution was poured on the native gel. The β-xylosidase and α-l-arabinofuranosidase activity bands were observed under UV light using gel documentation system (Gene Genius, Cambridge, UK). Esterase activity in PAGE gels was detected using 4-methylumbelliferyl acetate (5 mM) as substrate. The substrate solution was prepared in dimethyl sulfoxide. Upon completion of electrophoresis, the gel was incubated in phosphate buffer (0.1 M, pH 6.0) for 30 min and then the substrate solution was poured on the gel and esterase bands were observed UV light using gel documentation system (Gene Genius, Cambridge, UK) (Sonia et al. 2006).

### Two-dimensional electrophoresis and protein identification

The enzyme extracts were desalted and concentrated using ultra-filtration Amicon cell fitted with PM-10 membrane (10 kDa cut off). Protein (150 μg) samples were loaded by passive in-gel rehydration at 20 °C in 125 μl rehydration buffer (8 M urea, 2 % CHAPS, Destreak reagent, 1 % IPG buffer pH 3–5.6 and 0.005 % bromophenol blue in Milli Q grade sterilized water). The IPG strips (7 cm) were rehydrated for 16 h at room temperature in rehydration buffer. The isoelectric focusing (IEF) was performed using Ettan IGPhor 3 system (GE, Healthcare Biosciences) in a stepwise manner using voltage hour program that increased linearly in following steps: 100 V, 2 h; 300 V, 2 h; 1000 V, 2 h; 5000 V, 3 h (gradient); 5000 V, 6 h (step), with a total of 51,000 Vh. Prior to SDS-PAGE, the IPG strips were incubated for 15 min in 6 ml of 0.05 M Tris–Cl (pH 8.8), 8 M urea, 30 % (v/v) glycerol, 2 % (w/v) SDS, 60 mM Dithiothreitol (DTT) and traces of bromophenol blue followed by incubation for 15 min in the same buffer except that DTT was replaced with 50 mM iodoacetamide. The equilibrated IPG strips were transferred onto 12 % polyacrylamide gels without stacking gel and overlaid with molten low melting agarose (0.5 %). The second dimension was run at constant of 25 mA. The electrophoresis was carried out using a Hoefer mini VE system (GE, Healthcare Biosciences) and the gels were stained using silver nitrate (Sharma et al. 2011). The protein spots (silver nitrate stained) resolved by 2DE/SDS-PAGE were excised from the gels for peptide mass fingerprinting (PMF). The PMF of the samples was carried out at TCGA (The Centre for Genomic Application, New Delhi) using LC/MS (Agilent 1100 series 2D Nano LCMS). Mass spectrometry data were compared with NCBI and Swiss Prot databases using the Mascot search algorithm.

### Effect of added xylanase (*M. flava)* on saccharification and subsequent fermentation of agro-residues into to ethanol

Different pretreated agro-residues (1 % H_2_SO_4_), rice straw, cotton straw, wheat straw (Kindly supplied by DBT-IOC Center for Advanced Bioenergy Research, Faridabad, India) carrot grass and alkali pretreated (1 % NaOH) carrot grass, rice straw (Mahajan et al. 2014) were hydrolysed at substrate loading rate of 15 % w/v in two different sets in 25-ml glass vials containing 0.75 g of the substrate. One set of the reactions comprised of Novozyme 22086, a cellulase complex containing 1000 BHU (biomass hydrolysing U/g enzyme) was added at 30 mg protein/g substrate, in addition β glucosidase Novozyme 22118 (15 CBU/g substrate) and xylanase derived from *M. flava* (300 U/g substrate) were also added. The volume of the reaction mixture was made to 5.0 ml with 50 mM sodium citrate buffer (pH 6.0). The second set was same as first except for no additional xylanase was added. The flasks were kept for 72 h at 50 °C and 170 rpm. After 72 h hydrolysate was subjected to two-stage fermentation. In first stage, the hydrolysates were inoculated with actively growing *Saccharomyces cerevisiae* (24-h old grown in GYE broth) @ 5 % v/v and incubated for 48 h at 30 °C and 80 rpm. Thereafter, the fermentation broth was inoculated with *Pichia stipitis* (24-h old grown in XYE broth) @ 5 % v/v for 48 h at 30 °C. Prior to addition of *P. stipitis* the flasks were kept at 50 °C for 6 h for inactivation of *S. cerevisiae* (Li et al. 2011). Ethanol content in the fermented broth was estimated using spectrophotometric method (Caputi et al. 1968).

## Results and discussion

### Xylanase production by *Malbranchea flava*

*Malbranchea flava* isolated from composting soil was found to utilize different complex carbon sources mainly derived from agro-residues for xylanase production. Of the different carbon sources used for culturing *M. flava,* sorghum straw supported maximal xylanase (7091 U/gds) production followed by rice straw (3700 U/gds) and paper waste (1421 U/gds). The structural complexity of cellulose/hemicellulose complex in these carbon sources plays an important role in differential induction of glycosyl hydrolases (Badhan et al. 2007; Soni et al. 2010). Sorghum straw, a cheap and readily available agro-based carbon source, previously found to induce high levels of xylanase in thermophilic fungus *Thermomyces lanuginosus* (Sonia et al. 2005) was selected for further studies. Studies on combination of sorghum straw with different media types showed that culture medium containing yeast extract as nitrogen source, KH_2_PO_4_, K_2_HPO_4_, MgSO_4_ and citric acid was most suitable (Sonia et al. 2005). Replacing yeast extract with casein as nitrogen source resulted in enhanced xylanase production as also observed in *Penicillium canescens* (Antonie et al. 2010). Xylanase production was further increased when 2 ml spore suspension (2.5 × 10^7^ spores/ml) was used as inoculum. Higher inoculum levels, however, resulted in decreased xylanase production, similar observation has been made by Markus et al. (1965) who observed that higher inoculum levels led to decreased production of enzyme whereas the growth rate was higher with the larger inoculum. The variables like temperature and pH did not alter the obtained xylanase levels significantly (data not shown). Optimization of process parameters using OFAT resulted in 8300 (U/g DW substrate). In addition, culture extract contained β-xylosidase (3.2 U/gds), α-l-arabinofuranosidase (1.18 U/gds) and acetyl esterase (11.28 U/gds).

### Process optimization of xylanase and debranching enzymes by *M. flava*

On the basis of preliminary studies, nitrogen concentration (casein), inoculum age and inoculum level were chosen as independent variables to study their effect on production of xylanase, β-xylosidase, α-l-arabinofuranosidase and acetyl esterase employing Box–Behnken design of experiments using response surface methodology. Response surface methodology has previously been employed as statistical tool for optimization of xylanase production by thermophilic fungal strains of *T. lanuginosus, Melanocarpus albomyces* IIS68, *Scytalidium thermophilum* and *Myceliophthora* sp. (Narang et al. 2001; Sonia et al. 2005; Kaur et al. 2006; Badhan et al. 2007). The results of these experiments provide valuable information regarding the existence of interactions and synergies between factors as well as identify the factors that influence the production of enzymes most significantly. Box–Behnken design for xylanase production and its debranching enzymes by *M. flava* is given in Table 1. The highest xylanase production (16,390 U/gds) was achieved at 3.5 % casein concentration, 36 h of inoculum age and 2 ml inoculum level. The lowest xylanase production (9700 U/gds) was achieved at 0.5 % casein concentration, 0 h of inoculum age (spore suspension) and 3 ml inoculum level. Response data were analyzed for linear, two-factor interaction (2FI), quadratic and cubic models. Sequential model sum of squares predicted that quadratic model best fits the response data (*R* ^2^ = 0.93) and model *lack of fit* is non-significant. Finally, quadratic model was built by eliminating non-significant model terms using backward elimination method. 2FI for casein concentration × inoculum level along with square terms for casein concentration and inoculum age were significant model terms with high *p* values (Table 2). The deduced quadratic model was highly significant at *p* value <0.0001 (*F* = 33.34, *d*_f_ = 6) and non-significant *lack of fit* (*p* = 0.9237) (Table 2). The model has CV of 4.1 % and predicted *R* ^2^ = 0.8893 is comparable to adjusted *R* ^2^ = 0.9238. The experimental data passed the normality test and actual versus predicted response (Fig S1). Thus, model can be navigated in the design space using model regression equation (Eq. 2).

2 $$Y = 2494.130 + 7381.681A + 112.335B + 1668.750 \times C - 1085.000A \times C - 811.461 \, A^{2} - 1.086 \, B^{2}$$

where *Y* (xylanase, U/gds), *A* (casein, %), *B* (inoculum age, h) and *C* (inoculum level, ml) are in actual levels.

**Table 2:** ANOVA table showing regression analysis for xylanase and xylan-debranching accessory enzymes produced by *M. flava*

Xylanase			β-xylosidase		
Source	*F* value	*p* value Prob. > *F*	Source	*F* value	*p* value Prob. > *F*
Model	33.34	<0.0001*	Model	47.92	<0.0001*
A-Casein (%)	44.80	<0.0001*	A-Casein (%)	56.42	<0.0001*
B-Inocul. age (h)	38.67	<0.0001*	B-Inocul. age (h)	37.23	0.0001*
C-Inocul. level (ml)	6.45	0.0294*	C-Inocul. level (ml)	12.41	0.0055*
AxC	33.99	0.0002*	AxB	32.49	0.0002*
A^2^	45.15	<0.0001*	A^2^	125.20	<0.0001*
B^2^	26.86	0.0004*	B^2^	18.03	0.0017*
Lack of fit	0.27	0.9237^#^	Lack of Fit	0.43	0.8295^#^
	*R* ^2^	0.952		*R* ^2^	0.966
	Adj. *R* ^2^	0.923		Adj. *R* ^2^	0.946
	Pred *R* ^2^	0.889		Pred *R* ^2^	0.902
	C.V. %	4.10		C.V. %	5.03

Acetyl esterases			L α Arabinofuranosidase		
Model	35.63	<0.0001*	Model	18.08	0.0001*
A-Casein (%)	227.04	<0.0001*	A-Casein (%)	22.49	0.0003*
B-Inocul. age (h)	16.94	0.0034*	B-Inocul. age (h)	13.67	0.0024*
C-Inocul. level (ml)	2.36	0.1631*	Lack of Fit	0.65	0.7339^*#*^
AxB	4.41	0.0689*			
BxC	6.48	0.0344*			
A^2^	8.24	0.0208*			
B^2^	14.32	0.0054*			
C^2^	5.62	0.0452*			
Lack of fit	0.52	0.7276^#^			
	*R* ^2^	0.972		R^2^	0.72
	Adj. *R* ^2^	0.945		Adj. R^2^	0.681
	Pred *R* ^2^	0.916		Pred R^2^	0.601
	C.V. %	5.71		C.V. %	11.00

* Significant at *p* < 0.05

^#^Not-significant at *p* < 0.05

The regression equation indicated that casein concentration and inoculum level as most significant interaction. Casein concentration has synergistic effect on xylanase production when casein concentration is at higher level (3.5 %) and inoculum level is at minimum level (2 ml). The optimized process condition for inoculum age is predicted at 60 h to maximize xylanase production. Previous statistical model-based studies have shown inoculum age and inoculum level as important process parameter in the production of xylanase under SSF by *T. lanuginosus, Aspergillus terreus*, respectively (Sonia et al. 2005; Laxmi et al. 2009). It has also been observed that the choice of nitrogen source is critical for obtaining higher levels of xylanase production under solid-state fermentation (Gomes et al. 1993; Singh et al. 2000). In present study casein as nitrogen source was found to support high xylanase production by *M. flava.* Casein has also been found to be optimal nitrogen source for production of xylanase in *Cyathus stercoreus, Penicillium canescens* (Saxena et al. 1994; Antonie et al. 2010).

**Fig. 1:**
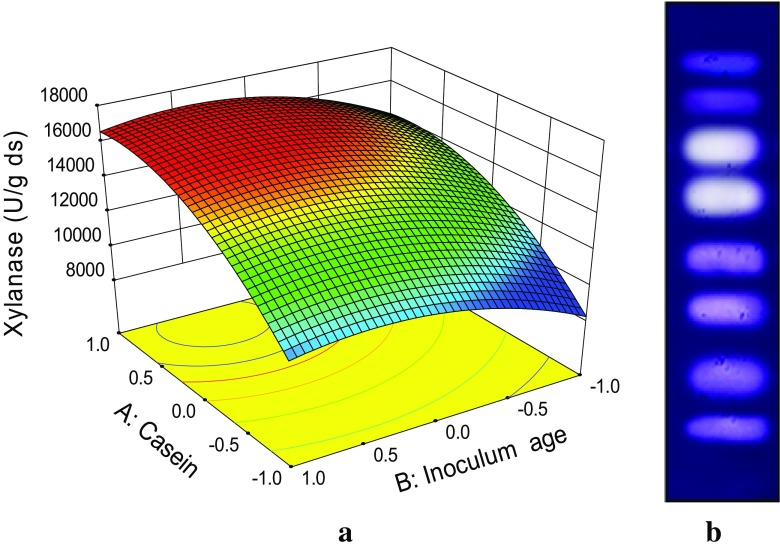
**a** 3D contour plot showing the effect of two-way interaction between casein concentration and inoculum age on xylanase production by *M. flava.* **b** Zymogram developed against culture extract proteins resolved by native PAGE indicating multiple xylanase isoforms

The level of xylanase (16,978 U/gds) produced under optimal culture conditions by *M. flava* (Fig. 1a) is only next to xylanase titers obtained by thermophilic fungal strains *T. lanuginosus* (48,000 U/gds) and *Paecilomyces thermophila* (18,580 U/gds) (Sonia et al. 2005; Yang et al. 2006). The culture under optimal conditions produced 8 distinct xylanase isoforms as revealed by native PAGE (Fig. 1b**)**. However, 6 distinct xylanase isoforms were resolved by IEF (Table 3). These included two major xylanases (pI 3.7 and 4.5) that have been previously purified and reported from this lab as thermostable xylanases of GH 11 and GH 10 families, respectively (Sharma et al. 2010). The xylanases corresponding to pI 4.0, 4.3, 4.8 and 5.0 were putatively designated as minor xylanases on the basis of observed lower intensity of xylanase active bands in zymogram. The xylanases resolved by IEF were eluted from the gel and were characterized for their substrate specificity against different xylan types birch wood xylan, wheat arabinoxylan, rye arabinoxylan, and methyl glucuronoxylan. The results showed xylanase corresponding to pI 4.5 (GH 10) showed similar activity against different xylan types whereas, xylanase (pI 3.7) GH 11 showed differential substrate specificity against BWX < WAX < RAX and MGX in that order. Thermophilic fungi are known to produce functionally diverse multiple xylanases as reported in *Myceliophthora* sp. (Badhan et al. 2004) and *Melanocarpus albomyces* (Saraswat and Bisaria 1997), previously. These diverse xylanases possibly give the producing cultures an advantage to utilize a variety of hemicellulosic heteropolymeric substrate that differ not only in their composition but also spatial distribution of sugar moieties.

**Table 3:**
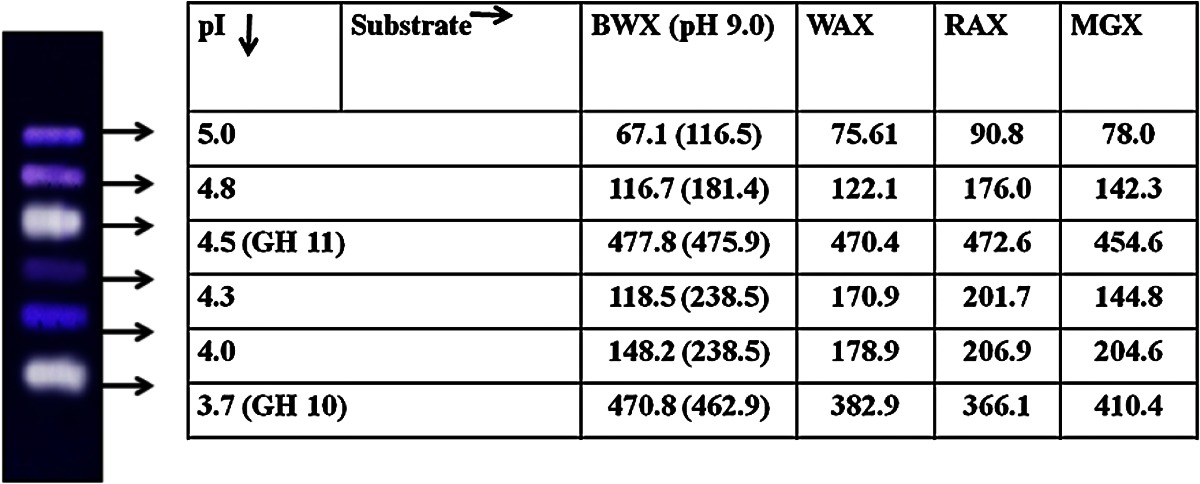
Functional characterization of multiple xylanases resolved on the basis of pI, against different xylan types

*BWX* Birchwood xylan, *WAX* Wheat arabinoxylan, *RAX* Rye arabinoxylan, *MGX* Methyl-Glucuronoxylan. The values shown are the specific activity of xylanase (units/mg protein)

#### β-Xylosidase

The highest β-xylosidase production (10 U/gds) was achieved at 2 % casein concentration, 72 h of inoculum age and 2 ml inoculum level (Table 1); whereas, the lowest β-xylosidase production (5.31 U/g DW substrate) was achieved at 3.5 % casein concentration, 0 h of inoculum age and 3 ml inoculum level. The fitted ANOVA model was significant at 99.99 % at *F* = 47.92, *df* = 6 with non-significant *lack of fit* at *p* = 0.8295 (Table 2). The fitted model explained 96.64 % variance (*R* ^2^ = 0.966) and CV of 5.03 %. Data passed the normal probability test and actual *vs.* predicted plots (*R* ^2^ = 0.966) (Fig S2). The fitted model equation in terms of actual process conditions is given as Eq. (3).

3 $$Y = 5.55161 + 3.929A + 0.028B - 0.512C + 0.021A \times B - 0.995A^{2} - 6.560 \times 10^{ - 4} B^{2}$$

where *Y* (β-xylosidase, U/gds), *A* (casein, %), *B* (inoculum age, h) and *C* (inoculum level, ml) are in actual levels.

**Fig. 2:**
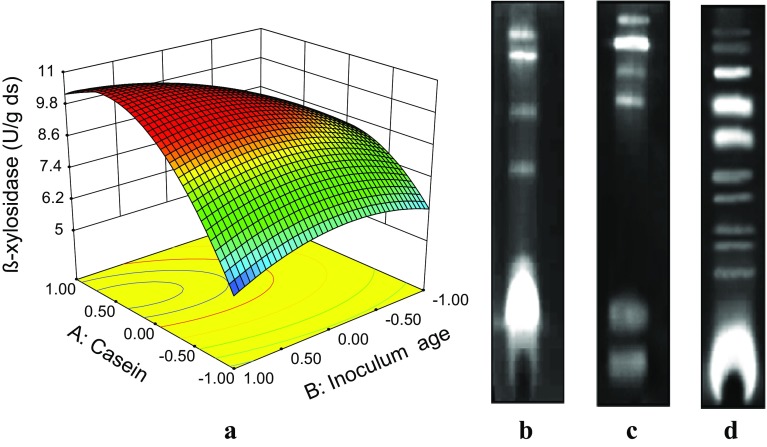
**a** 3D contour plot showing the effect of two-way interaction between casein concentration and inoculum age on β-xylosidase production by *Malbranchea flava* under solid substrate fermentation. **b** Activity staining of native PAGE gel showing β-xylosidase **c** α-l-arabinofuranosidase and **d** acetyl esterase isoforms

Two-factor interaction between casein concentration and inoculum age significantly affected β-xylosidase production. The interaction affect was most significant when casein concentration is at 3.5 % and inoculum age is around 60 h. However, maximal β-xylosidase expression is more favorable around 2.5–3.0 % casein concentration (Fig. 2a). The culture under optimized conditions produced 5 β-xylosidase isoforms of distinct electrophoretic mobility with one being major and other 4 being minor bands of low intensity (Fig. 2b). The observed β-xylosidase titre produced by *M. flava* is much higher to that observed in thermophilic fungus *T. lanuginosus* (Gomes et al. 1993) and comparable to those observed in *T. reesei* (Xin et al. 2010).

#### α-l-Arabinofuranosidase

The highest level of arabinofuranosidase (3.60 U/gds) was obtained when casein concentration (3.5 %) and inoculum level (4 ml) were at maximum levels and the inoculum age was 36 h (Table 1). Visual diagnostic passed normal probability plot along with actual *vs.* predicted plots (Fig S3). Fitted models of ANOVA resulted in reduced linear model as best fitted model with non-significant lack of fit (Table 2). Maximal expression of α-l-arabinofuranosidase of 3.67 (U/g DW substrate) could be achieved when inoculum age was between 50 and 55 h and casein concentration was 3.2–3.5 %. The linear fitted model equation is given as Eq. (4).

4 $$Y = 1.818 + 0.366 \, A + 0.011 \, B$$

where *Y* (arabinofuranosidase, U/gds), *A* (casein, %), *B* (inoculum age, h) and *C* (inoculum level, ml) are in actual levels.

The culture under optimal conditions produced six arabinofuranosidase isoforms. Four of these isoforms were common with those observed in the β-xylosidase zymogram (Fig. 2c). The major intensity bands in both the gels, however, differed but they both showed possible cross specificity for arabinose and xylose. The spatial similarity between xylopyranose and arabinofuranoside leads to bi-functional β-xylosidase/α-l-arabinofuranosidase found mainly in GH 3, 43 and 54 families (Mai et al. 2000). The observed distinct α-l-arabinofuranosidases may have role in efficient hydrolysis of arabinoxylans, where α-l-arabinofuranosidase (GH 43) releases *O*-3 linked arabinofuranosyl residues from double-substituted xylose in contrast, α-l-arabinofuranosidase (GH 62) that releases *O*-2- or *O*-3-linked arabinofuranosyl from mono-substituted xylose (Hinz et al. 2009).

#### Acetyl esterases

The fitted model for acetyl esterase (AE) production was significant and can be used for model predictions. Normal probability plot and actual versus predicted plot (*R* ^2^ = 0.972) (Fig S4) indicated that model can be navigated in the design space. The fitted model of actual process conditions is given as Eq. (5). Casein concentration is single most significant process variable in the production of acetyl esterases. On the other hand, 2FI (figures not shown) between casein concentration × inoculum age and inoculum age × inoculum level were significant interactions (Table 2). Under optimized conditions, 66.6 (U/g DW substrate) of acetyl esterases can be obtained at 3.5 % casein concentration, 52.5 h inoculum level and 2.30 ml inoculum level. The culture under optimal conditions produced multiple AEs with one of major bands showed high electrophoretic mobility (Fig. 2d). These functionally distinct multiple acetyl xylan esterases are essentially required for removal of acetic acid that esterifies the xylose units at *O*-2 and *O*-3 position (Sonia et al. 2006; Hinz et al. 2009)

5 $$Y = 64.194 + 14.995A{-}5.856 \times 10^{ - 3} B - 24.570C + 0.054 \, A \times B + 0.099B \times C - 1.746 \, A^{2} - 3.996 \times 10^{ - 3} B^{2} + 3.245C^{2}$$

where *Y* (acetyl esterases, U/gds), *A* (casein, %), *B* (inoculum age, h) and *C* (inoculum level, ml) are in actual levels.

The titres of xylan-debranching accessory enzymes (β-xylosidase, α-l-arabinofuranosidase and acetyl esterase) were expectedly much less when compared to xylanase activity observed in *M. flava*. Similar observations have also been made in different strains of *T. lanuginosus* (Singh et al. 2000). Singh et al. (2000) concluded that very high folds of extracellular xylanase levels relative to the accessory enzyme levels produced on various substrates may be inherent to many hyper-xylanase-producing fungal strains.

The observed experimental data was validated by performing the experiments in replicates at different time intervals and the results were found to be in agreement with the proposed model. Under optimized culture conditions (3.0–3.5 % casein concentration, 50–60 h inoculum age and 2–2.5 ml inoculum level) production of hemicellulolytic enzyme activities of xylanase (16,978 ± 102 U/gds), β-xylosidase (10.0 ± 0.55 U/gds), α-l-arabinofuranosidase (3.80 ± 0.21 U/gds) and acetyl esterase (67.7 ± 2.1 U/gds) was achieved. The optimization studies carried out resulted in 2.04-, 3.1-, 3.2- and 6.0-folds increase in the levels of xylanase, β-xylosidase, α-l-arabinofuranosidase and acetyl esterase (AE) activities, respectively, when compared to enzyme optimized condition by OFAT.

### Electrophoretic profiling of secretome of *Malbranchea flava*

**Fig. 3:**
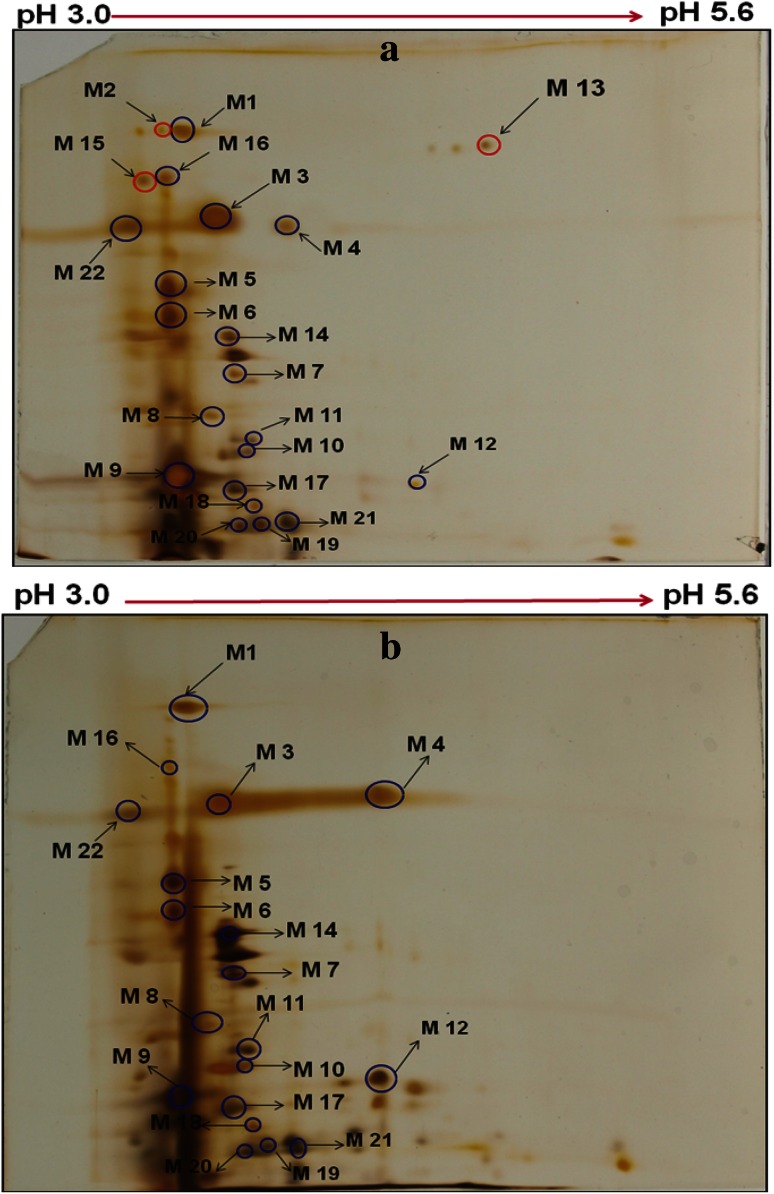
2-DE gel showing proteins expressed in presence of **a** basal medium, **b** optimized medium. Protein spots marked in *blue* were expressed in presence of both basal and optimized medium, whereas, protein spots marked in *red* were absent in optimized medium

**Fig. 4:**
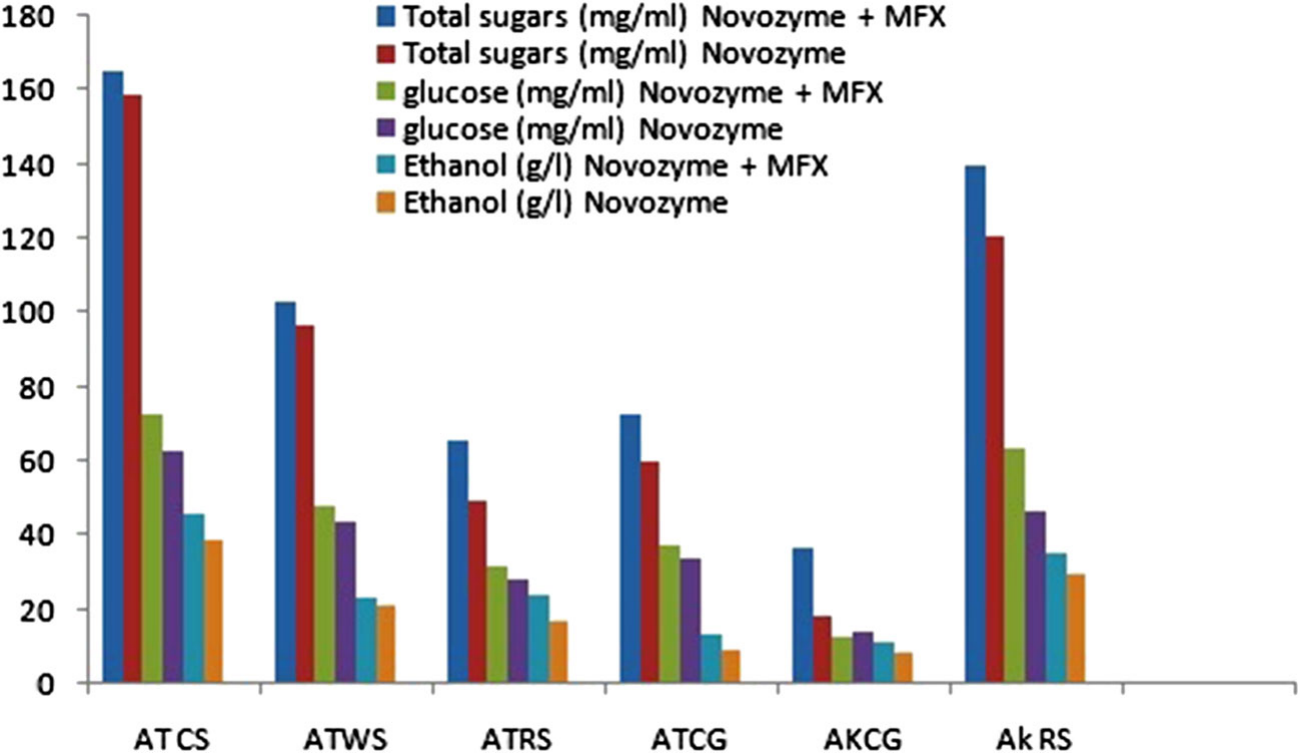
Effect of supplementation of xylanase from *M. flava* to the commercial cellulase in enhancing saccharification and subsequent fermentation of hydrolysate into ethanol (ATCS, acid treated cotton stalks; ATWS, acid treated wheat straw; ATRS, acid treated rice straw; ATCG, acid treated carrot grass; AKCG, alkali treated carrot grass; AkRS, alkali treated rice straw) Y axis denoted sugar (mg/ml) and ethanol concentration (g/l)

*Malbranchea flava,* grown on solidified sorghum straw medium under unoptimized and optimized culture conditions was compared for secretome expression profiles. The crude enzyme extracts, prepared as described in “Materials and methods”, were resolved by 2DE (Fig. 3a, b). Seventy-two protein spots were detected in the pI range of 3.0–5.6 when the culture was grown on sorghum straw under optimal culture conditions. The culture grown under two conditions showed distinct secretome profile that showed differences in the expression intensity and profile (Fig. 4; Table 4). It was observed that the expression of eight protein spots (M1, M3, M4, M7, M10, M11, M12 and M14) was up-regulated when the culture was grown under optimal conditions as evident from the intensity of spots obtained (Fig. 4b). However, the protein spots M9 and M22 were down regulated under optimal culture conditions. On the other hand, it was observed that the proteins spots M2, M13 and M15 were expressed in the secretome of *M. flava* grown under unoptimized conditions were not detected under the optimal culture conditions (Fig. 4a, b). Ten protein spots resolved by 2DE were identified using LC/MS–MS approach (Table 4). On the basis of homology search (Swiss Prot), the spot M3 and M4 (Fig. 4a) were identified as two distinct β-1, 4-xylanases belonging to GH-11 family. Protein spots M5, M6, M15 and M16 were identified as cellobiose dehydrogenase (CDH) indicting to presence of oxidative cleaving capability of *M. flava secretome*. CDH is known to act as electron donor for AA9 (auxiliary enzymes) to bring about efficient hydrolysis of crystalline regions of cellulose (Horn et al. 2012). The protein spot M9 was identified as unreported putative cellobiohydrolase 6 (GH 6). In addition, two hypothetical proteins M1 and M14 were also identified in the secretome of *M.* *flava*. Previous studies reporting comparative secretome of the fungal strains grown under submerged and solid-state fermentation (Oda et al. 2006) between different hyper-secretory strains of *T.* *reesei* or those grown in presence of different carbon sources also highlighted differential expression profiles resulting in identification of unreported putative endoglucanases and arabinofuranosidases (Gimbert et al. 2008).

**Table 4:** Identification of the protein spots shown in Fig. 3 *M. flava* secretome by LC–MS/MS

Sample	Mascot score	Identified protein	Query peptide matches	Matched peptides	Closest relative (NCBI Non redundant proteins)	*E* value
M1	51	Hypothetical protein	2	R.DIVSALTRDVK.Q	*Chaetomium globosum* XP001223857.1	0.004
M3	230	β-1,4 xylanase (GH 11)	14	R.ITVADVG.-R.SSGTVQTGCHFDAWAR.A R.AGLNVNGDHYYQIVATEGYFSSGYAR.I	*Paecilomyces* sp. ACS26244.1 *T. lanuginosus* ACS769861.1.	4e^−18^ 4e^−18^
M4	61	β-1,4 xylanase ((GH 11)	9	R.ITVADVG.-R.SSGTVQTGCHFDAWAR.A	*Paecilomyces* sp. ACS26244.1 *T. lanuginosus* ACS769861.1.	4e^−08^ 2e^−18^
M5	77	Cellobiose dehydrogenase	2	R.VILSAGTFGTPK.I	*Aspergillus clavatus* XP001276037.1	8e ^−04^
M6	140	Cellobiose dehydrogenase	3	R.DGGTAVVDLNTK.V R.VILSAGTFGTPK.I	*Aspergillus clavatus* XP001276037.1	1e ^−05^
M9	59	Hypothetical protein MGG004499	4	R.DGVAYALK.T	*Magneporthe oryzae* XP362054.1 *Pyrenophora tritici repentis* XP0019316323.1	3.2 25
M12	50	Mitochondrial and cytoplasmic glycyl-tRNA synthase	1	K.EEYEQILAK.I	*Pichia pastoris* XP002492599.1	0.070
M14	54	Hypothetical protein	1	R.IELSEVQR.V	*Trichophyton verrucosum* HKI0517 XP003025276.1	1.3
M15	150	Cellobiose dehydrogenase	2	R.DGGTAVVDLNTK.V R.VILSAGTFGTPK.I	*Aspergillus clavatus* XP001276037.1	1e ^−05^
M16	135	Cellobiose dehydrogenase	2	R.DGGTAVVDLNTK.V R.VILSAGTFGTPK.I	*Aspergillus clavatus* XP001276037.1	1e ^−05^

### Effect of *M. flava* xylanase on hydrolysis

The results (Fig. 4) show that hydrolysis of acid pretreated cotton stalk resulted in comparatively higher release of total sugars, glucose and ethanol followed by alkali-treated rice straw and acid-treated wheat straw. Addition of xylanase to the commercial enzyme resulted in appreciably higher levels of hydrolysis ranging between 9 and 36 % improved release of glucose when compared to hydrolysis with commercial enzyme only. Subsequent fermentation of hydrolysates with co-culture of *S. cereviase* and *P stipitis* (Li et al. 2011) resulted in maximal ethanol (46 g/l) in acid-treated cotton stalk treated with supplemented xylanase as compared to 39 g/l obtained with commercial cellulase alone (Fig. 4). The results clearly suggest the positive role of xylanases from *M. flava* in enhancing the hydrolysis that subsequently resulted in higher ethanol yields in all the treated substrates tested in the study. Xylanase (GH10 and GH11) from *T. reesei* have also been shown to enhance the hydrolysis of different pretreated substrates, however, at much higher xylanase protein load (Hu et al. 2013). The observed improved level of hydrolysis in presence of *M flava* xylanases may be attributed to higher catalytic efficiency and thermostability of xylanases from thermophilic fungi that are able to maintain the activity during hydrolysis (Sharma et al. 2010).

## Conclusions

Thermophilic fungus *M. flava* was found to produce high levels of xylanase under SSF using sorghum straw, a cheap and readily available carbon source. The xylanases from *M. flava* characterized as thermostable and catalytically efficient from this lab (Sharma et al. 2010) demonstrated the potential in improving bioconversion of lignocellulosics into ethanol. The culture is being further developed into commercially important source of xylanase through strain development approaches.

## Electronic supplementary material

Below is the link to the electronic supplementary material.

Supplementary material 1 (DOC 53 kb)

Supplementary material 2 (DOCX 11 kb)
