# Supplementary material for: Profiling and production of hemicellulases by thermophilic fungus Malbranchea flava and the role of xylanases in improved bioconversion of pretreated lignocellulosics to ethanol
Source: 3 Biotech. 2016 Jan 14;6(1):30. doi: 10.1007/s13205-015-0325-2 (PMC4713398; doi:10.1007/s13205-015-0325-2)
Supplement: Supplementary file 1 — Supplementary material 1 (DOC 53 kb) [file 13205_2015_325_MOESM1_ESM.doc]

|  | a |
| --- | --- |
|  | b |

**Fig. S1**a:

|  | a |
| --- | --- |
|  | b |

**Fig. S1 b**:

|  | a |
| --- | --- |
|  | b |

**Fig. S1 c**:

|  | a |
| --- | --- |
|  | b |

**Fig. S1d:**

**Supplementary Information (SI)**

**Fig. S1**: a) Normal plot of residuals b) actual *vs.* predicted response for xylanase

**Fig. S2**: a) Normal plot of residuals b) actual *vs.* predicted response for β-xylosidase

**Fig. S3**: a) Normal plot of residuals b) actual *vs.* predicted response for α-L-arabinofuranosidase

**Fig. S4**: a) Normal plot of residuals b) actual *vs.* predicted response for acetyl esterase
